# Prenatal ultrasound and postmortem histologic evaluation of tooth germs: an observational, transversal study

**DOI:** 10.1186/s13005-015-0075-8

**Published:** 2015-05-12

**Authors:** Mariana Seabra, António Felino, Rosete Nogueira, Francisco Valente, Ana Cristina Braga, Paula Vaz

**Affiliations:** Faculty of Dentistry, University of Porto (FMDUP), 4200-393 Porto, Portugal; Department of Oral Surgery of FMDUP, Porto, Portugal; Pathology Laboratory of CGC Genetics/Centro de Genética Clínica, Porto, Portugal; Prenatal Diagnosis Unit, Hospital of Vila Nova de Gaia/Espinho (CHVNG/E), Porto, Portugal; Department of Production and Systems Engineering, and researcher in Algoritmi Centre University of Minho, Braga, Portugal; Department of Medical and Orofacial Genetics of FMDUP, Porto, Portugal; Portuguese Catholic University (UCP), Viseu, Portugal; Life and Health Sciences Research Institute (ICVS), School of Health Sciences (ECS), Campus of Gualtar, University of Minho, Braga, Portugal; ICVS/3B’s - PT Government Associate Laboratory, Braga, Guimarães Portugal

**Keywords:** Tooth germ, Tooth buds, Ultrasound, Prenatal, Diagnosis, Histology

## Abstract

**Introduction:**

Hypodontia is the most frequent developmental anomaly of the orofacial complex, and its detection in prenatal ultrasound may indicate the presence of congenital malformations, genetic syndromes and chromosomal abnormalities.

To date, only a few studies have evaluated the histological relationship of human tooth germs identified by two-dimensional (2D) ultrasonography. In order to analyze whether two-dimensional ultrasonography of tooth germs may be successfully used for identifying genetic syndromes, prenatal ultrasound images of fetal tooth germs obtained from a Portuguese population sample were compared with histological images obtained from fetal autopsies.

**Methods:**

Observational, descriptive, transversal study. The study protocol followed the ethical principles outlined by the Helsinki Declaration and was approved by the Ethics Committee of the School of Dental Medicine, University of Porto (FMDUP, Porto, Portugal) and of the Centro Hospitalar de Vila Nova de Gaia/Espinho (CHVNG/EPE, Porto, Portugal) as well as by the CGC Genetics Embryofetal Pathology Laboratory. Eighty-five fetuses examined by prenatal ultrasound screening from May 2011 to August 2012 had an indication for autopsy following spontaneous fetal death or medical termination of pregnancy. Of the 85 fetuses, 37 (43.5%) were randomly selected for tooth germ evaluation by routine histopathological analysis. Fetuses who were up to 30 weeks of gestation, and whose histological pieces were not representative of all maxillary tooth germs was excluded. Twenty four fetus between the 13^th^ and 30^th^ weeks of gestation fulfilled the parameters to autopsy.

**Results:**

Twenty four fetuses were submitted to histological evaluation and were determined the exact number, morphology, and mineralization of their tooth germs. All tooth germs were identifiable with ultrasonography as early as the 13^th^ week of gestation. Of the fetuses autopsied, 41.7% had hypodontia (29.1% maxillary hypodontia and 20.9% mandibular hypodontia).

**Conclusions:**

This results indicate that prenatal ultrasound is a reliable method for detecting of hypodontia an early gestational ages. Further studies with larger samples are needed to confirm these results.

## Introduction

Hypodontia or tooth agenesis is the most frequent developmental malformation of the orofacial complex [1] and is commonly associated with other abnormalities in many syndromes [2,3]. Detection of hypodontia in prenatal ultrasound may therefore be a sign of congenital malformations, genetic syndromes and chromosomal abnormalities [4,5].

Human odontogenesis occurs over a long period, approximately from the 6th intrauterine week until late adolescence, when the roots of the third molars are formed [6]. In the mammalian embryo, teeth develop via a series of interactions between the odontogenic epithelium and the neural crest-derived ectomesenchyme of the early jaw [7-11].

**Figure 1:**
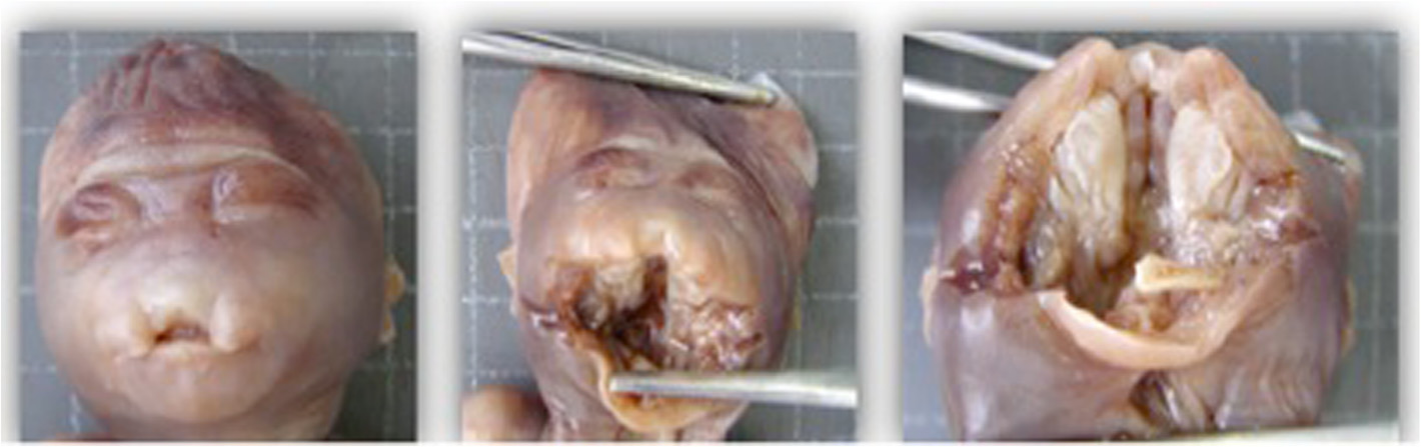
Fetus at 21 weeks GA with removal of the upper jaw and mandible. Source: CGC Genetics/Embryofetal Pathology Laboratory, Portugal.

Studies about intrauterine tooth development in humans are scarce and most work in this area to date has been conducted in rodents [12]. Although mouse models have been extremely valuable to gain a better understanding of human craniofacial development, rodent and human dental formulas diverge [13], making it difficult to extrapolate the results to our understanding of human dental development. Therefore, research with human fetuses is essential to expand our knowledge in this area (Figure 1). Fetal or perinatal autopsy is useful to determine different developmental parameters, to detect congenital abnormalities, to identify the cause of death and the risk of possible recurrence, and to identify possible genetic syndromes [14].

To date, only a few studies have evaluated the histological relationship of human tooth germs identified by two-dimensional (2D) ultrasonography. Most of these studies, conducted with a limited number of pregnant women and abortion products [4,5,15], failed to visualize tooth germs with ultrasonography during the early gestational stages and to identify all tooth germs in fetal autopsies. To analyze whether 2D ultrasonography was an adequate tool for evaluating tooth germs and identify genetic syndromes, our study investigated whether prenatal ultrasound images of fetal tooth germs obtained from a Portuguese population sample correlated with histological images obtained from fetal autopsies.

## Methods

The study protocol followed the ethical principles outlined by the Helsinki Declaration and was approved by the Ethics Committee of the Dental Medicine Faculty, University of Porto (FMDUP, Porto, Portugal), and of the Centro Hospitalar de Vila Nova de Gaia/Espinho (CHVNG/EPE, Porto, Portugal) as well as by the CGC Genetics Embryofetal Pathology Laboratory.

Fetal tooth germs were visualized with 2D prenatal ultrasound (GE E8 Voluson® equipment, serial number 0123, 2010, Austria) with C512D, Rab4-8D, 11LD, and C1-5 probes, with normal harmonic frequency. The images were viewed, captured, and archived, using the Astraia® program (version 1.23.0, Astraia Software Gmbh and parents, Germany), and processed on the same equipment with dimensions of 640 * 480 VGA pixels.

Two operators were reliable for conducting examinations and recording data. They were both specialists in fetal medicine and had equivalent levels of practice in obstetric ultrasound and prenatal diagnosis. Calibration was achieved on both operators to ensure a correct understanding of the ultrasound images.

Fetal tooth germs in both maxilla and mandible were visualized with 2D prenatal ultrasound (GE E8 Voluson® equipment) in a group of 157 pregnant Portuguese participants being seen at the CHVNG/EPE. Informed consent was obtained from participants before the exam.

Eighty-five fetuses examined by prenatal ultrasound screening from May 2011 to August 2012 had an indication for autopsy following spontaneous fetal death or medical termination of pregnancy. Of these, 37 (43.5%) were randomly selected for tooth germ evaluation by routine histopathological analysis. Of these 13 were excluded because they did not meet the study’s inclusion criteria, which were the following: 1) fetuses who were up to 30 weeks of gestation, and 2) fetuses whose histological pieces were not representative of all maxillary tooth germs. Therefore, the final sample included 24 autopsied fetuses who were between the 13^th^ and 30^th^ weeks of gestation (Table 1). Each fetus was examined according to a predesigned protocol from the Embryofetal Pathology Laboratory CGC Genetics, which included a photograph, a whole body radiograph, and external and internal examination and microscopic study. Prior to post-mortem examination, written consent was obtained from the father or from another relative.

**Table 1:** **Distribution of fetuses according to gestational ages (GA)**

**GA**	**N**	**%**
13	1	4.2
14	4	16.6
15	4	16.7
16	2	8.3
17	1	4.2
18	1	4.2
21	5	20.9
22	3	12.5
26	1	4.2
28	1	4.2
30	1	4.2
Total	24	100.0

The statistical analysis was performed using IBM® SPSS (Statistical Package for Social Sciences) version 22.0 and R version 2.15.1 (2012-06-22). Given the nature of the variables involved, it was decided to use statistical tools based on the analysis most appropriate to the scales of measurement. Thus, the analysis consisted of the prevalence study in which estimates were determined for all parameters evaluated, as well as interval estimates with 95% confidence, and analytical study of the data for qualitative variables, where the association between two variables was established using the chi-square test of independence (for 2x2 tables the exact test of Fisher was used). The decision rule consists of detecting statistically significant evidence for probability value (p-value of the test) less than 0.05.

## Results

For the 157 fetuses who were initially examined by 2D ultrasound, the median gestational age (GA) at which all 10 maxillary and mandibular tooth germs were identified was the 13th week. In 25% of cases, GA was equal to or less than 12 weeks.

Only fetuses from interrupted pregnancies or cases of fetal death were autopsied. The remaining clinical cases followed a normal pregnancy. We analyzed 37 cases of fetal death/pregnancy medical interruption. Of these, 13 were excluded because they did not meet the inclusion criteria.

Of the 24 autopsied fetuses (Table 1), 14 (58.3%) were male and 10 (41.7%) were female. The chi-square test revealed no significant differences related to gender distribution (χ^2^ = 0.75, df = 1, p value = 0.3865 > 0.05). Thirteen (13) of the fetuses were medical abortions (MA) and 11 were spontaneous abortions (SA). The MA cases were associated with relatively earlier gestational stages . A Fisher’s exact test revealed that spontaneous abortions were more frequent in male fetuses (p = 0.004).

**Figure 2:**
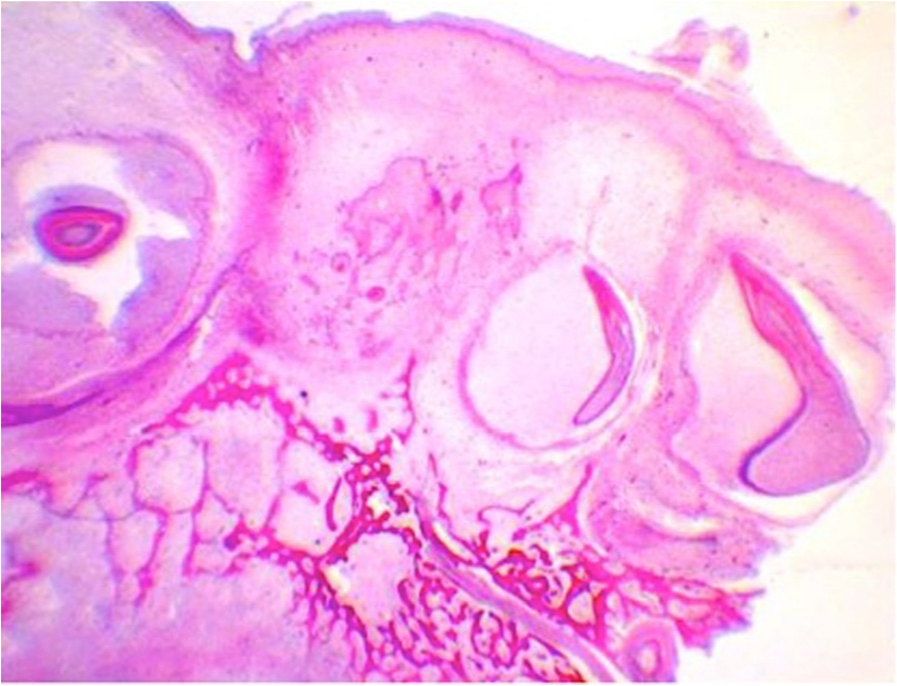
Histological section of maxilla from a fetus at 30 GA weeks. Initial mineralization of dental germs from temporary dentition (HE 10x). Source: CGC Genetics/Embryofetal Pathology Laboratory, Portugal.

**Figure 3:**
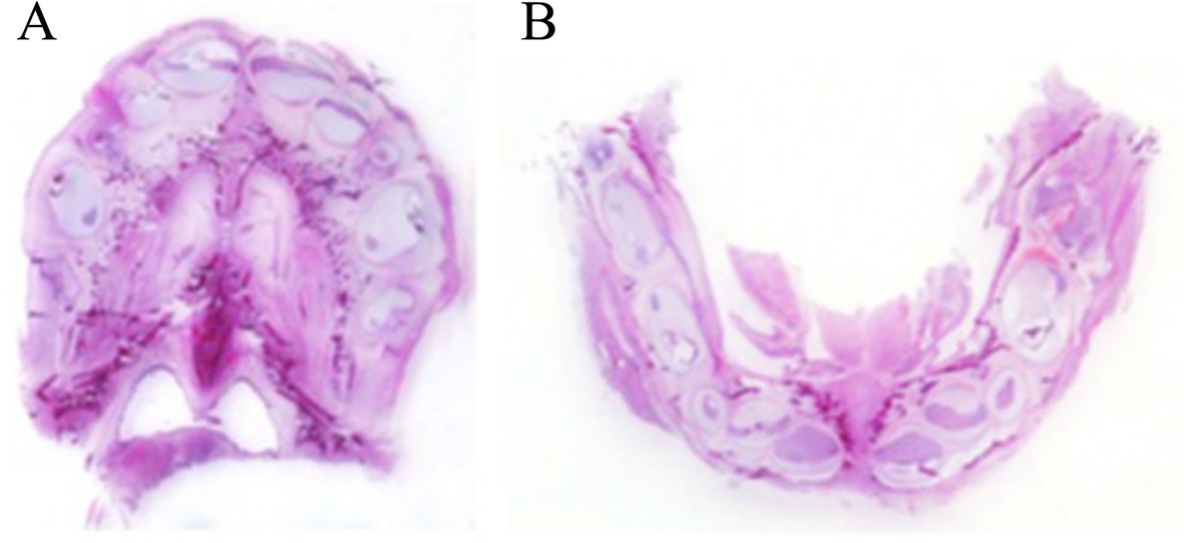
Fetus at 21 GA weeks. All temporary tooth germs are present at maxilla **(A)** and mandibula **(B)**; (HE macro). Source: CGC Genetics/Embryofetal Pathology Laboratory, Porto, Portugal.

**Figure 4:**
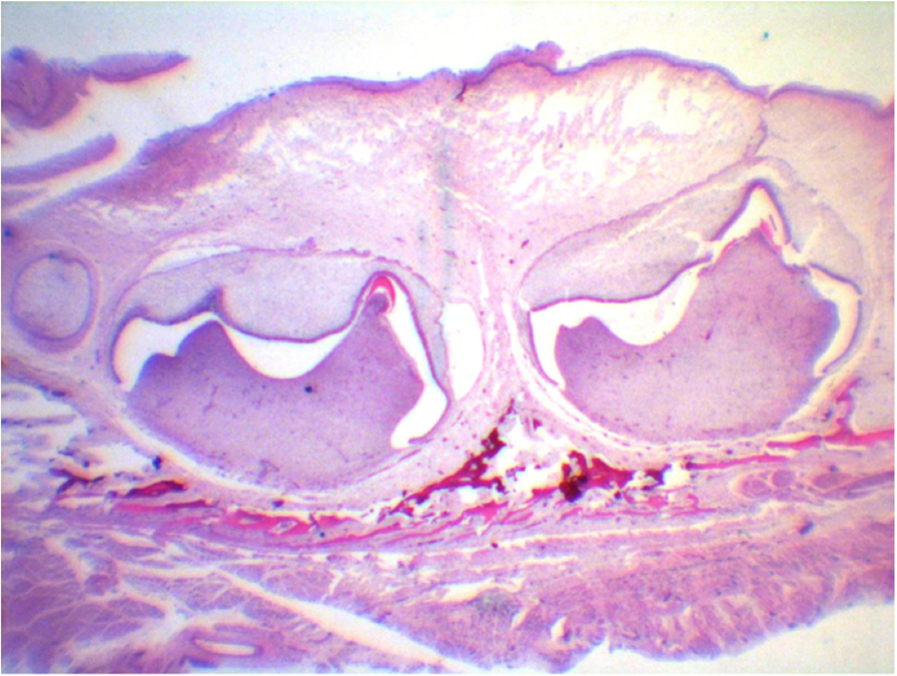
Histological section of jaw from fetus at 21 weeks GA – (HE 10x). Source: CGC Genetics/Embryofetal Pathology Laboratory, Portugal.

**Figure 5:**
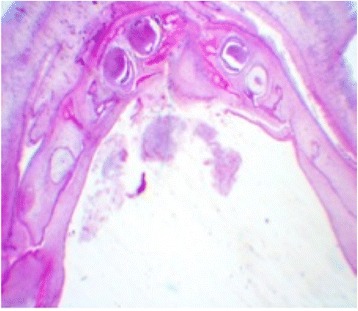
Histological section of a mandible from fetus at 15 weeks of GA. Hypodontia of temporary tooth germs (HE 10x).Source: CGC Genetics/Embryofetal Pathology Laboratory Porto, Portugal.

It was determined the exact number, morphology, and mineralization of tooth germs in the 24 fetuses who underwent histological evaluations (Figures 2, 3, and 4). All tooth germs were histologically identified during the 13th week of gestation. Of the 24 fetuses autopsied, 41.7% had hypodontia (Table 2) (Figure 5).

**Table 2:** **Prevalence of hypodontia in fetal autopsies**

		**N**	**Percent**	**95% Confidence interval**
**Valid**	**Absence**	14	58.3	-
	**Presence**	10	41.7	[22.8%; 3.1%]
	**Total**	24	100.0

Females and males were similarly affected by hypodontia (p = 0.132) (Table 3). Maxillary and mandibular hypodontia were observed in 29.1% and 20.9% of fetuses, respectively (Tables 4 and 5).

**Table 3:** **Hypodontia and fetus gender**

			**Gender**		**Total**
			**Female**	**Male**	
**Hypodontia**	**No**	Count	4	10	14
	**Yes**	Count	6	4	10
**Total**		Count	10	14	24

**Table 4:** **Maxillary hypodontia and clinical information**

			**Clinical information**		**Total**
			**MA**	**SA**	
**Maxillary hypodontia**	**No**	Count	9	8	17
	**Yes**	Count	4	3	7
**Total**		Count	13	11	24

**Table 5:** **Mandibular hypodontia and clinical information**

			**Clinical information**		**Total**
			**MA**	**SA**	
**Mandibular hypodontia**	**No**	Count	11	8	19
	**Yes**	Count	2	3	5
**Total**		Count	13	11	24

A Fisher’s exact test showed no significant association between maxillary (p = 1.000) or mandibular (p = 0.630) hypodontia and clinical information concerning the number of spontaneous or medical abortions (Tables 4 and 5).

The teeth most frequently missing in the maxilla were 65 and 55, while 81 was the most frequently missing tooth in the mandible.

## Discussion

**Figure 6:**
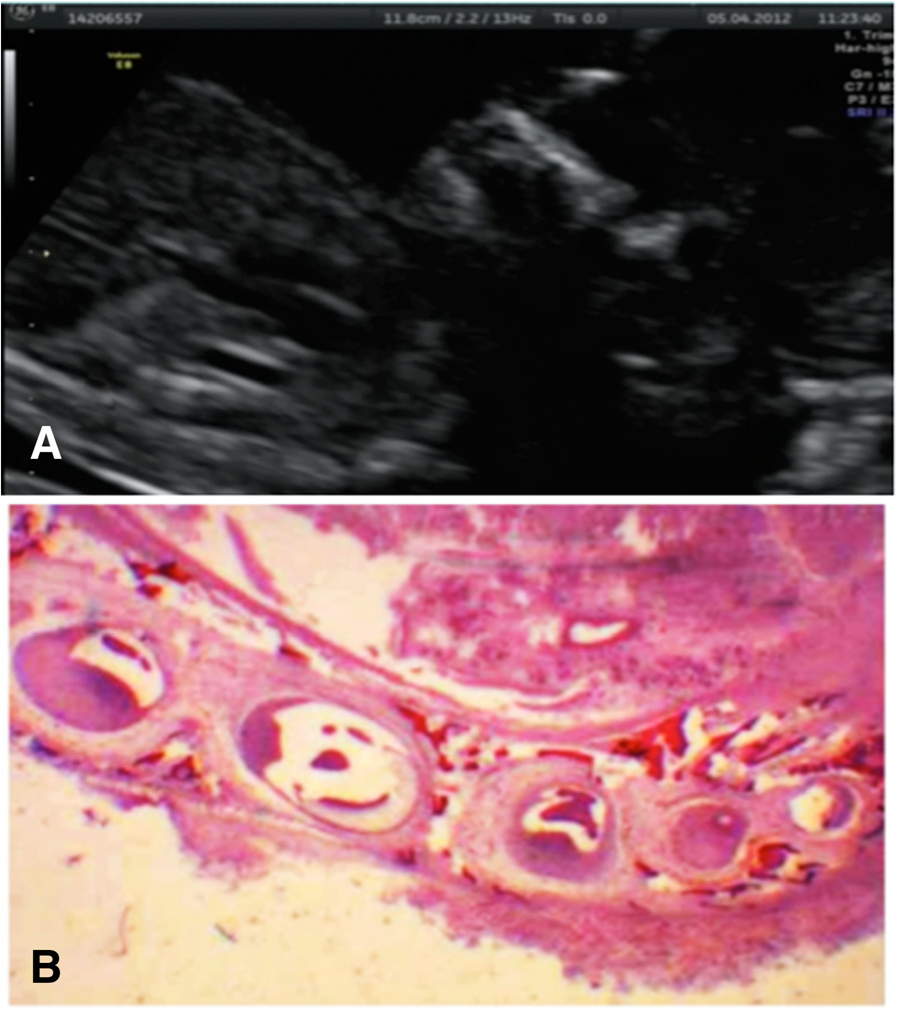
Ultrasound 2D image of fetus maxilla. Source: CHVNG/EPE **(A) (B)** Histological section of a maxilla from fetus at 13 weeks of GA. Source: CGC Genetics/Embryofetal Pathology Laboratory Porto, Portugal.

In this study, we showed that all tooth germs were histologically present at the 13th week of gestation, as revealed by prenatal ultrasonography (Figure 6). Therefore, we believe that visualization of tooth germs with 2D ultrasonography during the 13th week of gestation is a useful method of identifying genetic syndromes.

To our knowledge, this study was the first to investigate the occurrence of hypodontia in temporary dentition in autopsied fetuses. The number, morphology, and mineralization of the tooth germs were identified in all histological evaluations of our sample.

Although tooth agenesis is the most frequent developmental malformation of the orofacial complex, its prevalence varies considerably between generations and classes of teeth. Agenesis of primary teeth is very rare, occurring at a frequency of less than 1% [16-18].

Some cases of tooth agenesis occur independently of developmental defects in other organs and are referred to as non-syndromic. However, missing teeth are also observed in association with other malformations, most noticeably with cleft lip, with or without cleft palate [18]. We detected hypodontia in 41.7% of autopsied fetuses, a much higher prevalence than that described for other non-syndromic populations, which varied from 0.1 to 2.63% [19-28]. This value may be related to the fact that tooth agenesis is usually associated with a variety of syndromes. As our sample consisted of fetuses who had suffered spontaneous death or medical termination of pregnancy, it is reasonable to assume that this group would present a higher number of syndromes associated with hypodontia.

Although it has been reported that females are generally more affected by hypodontia than males [29], we did not detect significant gender differences in the autopsied fetuses evaluated in the current study. Further studies with larger samples are needed to determine whether the prevalence of hypodontia is associated with gender in aborted fetuses with congenital malformations and genetic syndromes. Whether a specific absence of tooth germs relates to some kind of fetal abnormality is another important question that remains unanswered.

## Conclusions

Our results indicate the reliability of the ultrasound method for the prenatal detection of hypodontia at early gestational ages. The assessment of tooth germs in prenatal ultrasound may allow for the early identification of genetic syndromes. We were able to visualize, identify, and count fetal tooth germs using prenatal 2D ultrasound, around the 13th week of gestation.

The early prenatal identification of tooth germs enables the diagnosis of genetic syndromes with tooth number abnormalities and may represent a new approach for the early intervention of these syndromes in pediatric dentistry. The early diagnosis of these genetic syndromes would allow de pediatric dentist to prepare for the correct interception treatment, which could occur at birth (oral cleft, applying the nasoalveolar molding and the oral plaque), during the first months of life or even after the third year of life.

## References

[1] Chhabra N, Goswami M, Chhabra A (2014). Genetic basis of dental agenesis - molecular genetics patterning clinical dentistry. Med Oral Patol Oral Cir Bucal.

[2] Matalova E, Fleischmannova J, Sharpe PT, Tucker AS (2008). Tooth agenesis: from molecular genetics to molecular dentistry. J Dent Res.

[3] De Coster PJ, Marks LA, Martens LC, Huysseune A (2009). Dental agenesis: genetic and clinical perspectives. J Oral Pathol Med.

[4] Ulm MR, Chalubinski K, Ulm C, Deutinger J, Plockinger B, Bernaschek G (1995). Sonographic depiction of fetal tooth germs. Prenat Diagn.

[5] Ulm MR, Kratochwil A, Ulm B, Lee A, Bettelheim D, Bernaschek G (1999). Three- dimensional ultrasonographic imaging of fetal tooth buds for characterization of facial clefts. Early Hum Dev.

[6] Townsend G, Bockmann M, Hughes T, Brook A (2012). Genetic, environmental and epigenetic influences on variation in humantoothnumber, size and shape. Odontology.

[7] Cobourne MT, Sharpe PT (2003). Tooth and jaw: molecular mechanisms of patterning in the first branchialarch. Arch Oral Biol.

[8] Jernvall J, Thesleff I (2000). Reiterative signaling and patterning during mammalian tooth morphogenesis. Mech Dev.

[9] Tucker AS, Sharpe P (2004). The cutting edge of mammalian development; how the embryo makes teeth. Nat Rev Genet.

[10] Tummers M, Thesleff I (2009). The importance of signal pathway modulation in all aspects of tooth development. J Exp Zool B: Mol Dev Evol.

[11] Cobourne MT, Sharpe PT (2010). Making up the numbers: The molecular control of mammalian dental formula. Semin Cell Dev Biol.

[12] Kapadia H, Mues G, D'Souza R (2007). Genes affecting tooth morphogenesis. Orthod Craniofac Res.

[13] Chai Y, Maxson RE (2006). Recent advances in craniofacial morphogenesis. Dev Dyn.

[14] Désilets V, Oligny LL (2011). Genetics Committee of the Society of Obstetricians and Gynaecology Canada; Family Physicians Advisory Committee; Medico–Legal Committee of the SOGC.Fetal and perinatal autopsy in prenatally diagnosed fetal abnormalities with normal karyotype. J Obstet Gynaecol Can.

[15] Ulm MR, Kratochwil A, Ulm B, Solar P, Aro G, Bettelheim D (1998). Three-dimensional ultrasound evaluation of fetal tooth germs. Ultrasound Obstet Gynecol.

[16] Arte S, Nieminen P. Hypodontia. [https://www.orpha.net/data/patho/GB/uk-hypodontia.pdf]

[17] Bailleul-Forestier I, Molla M, Verloes A, Berdal A (2008). The genetic basis of inherited anomalies of the teeth. Part 1: clinical and molecular aspects of non-syndromic dental disorders. Eur J Med Genet.

[18] Mitsiadis TA, Luder HU (2011). Genetic basis for tooth malformations: from mice to men and back again. Clin Genet.

[19] Brook AH (1974). Dental anomalies of number, form and size: their prevalence in British schoolchildren. J Int Assoc Dent Child.

[20] Magnusson TE (1984). Hypodontia, hyperodontia, and double formation of primary teeth in Iceland. An epidemiological study. Acta Odontol Scand.

[21] Jones ML, Mourino AP, Bowden TA (1993). Evaluation of occlusion, trauma, and dental anomalies in African-American children of metropolitan Headstartprograms. J Clin Pediatr Dent.

[22] Whittington BR, Durward CS (1996). Survey of anomalies in primary teeth and their correlation with the permanent dentition. N Z Dent J.

[23] Yonezu T, Hayashi Y, Sasaki J, Machida Y (1997). Prevalence of congenital dental anomalies of the deciduous dentition in Japanese children. Bull Tokyo Dent Col.

[24] Carvalho JC, Vinker F, Declerck D (1998). Malocclusion, dental injuries and dental anomalies in the primary dentition of Belgian children. Int J Paediatr Dent.

[25] Altug-Atac AT, Erdem D (2007). Prevalence and distribution of dental anomalies in orthodontic patients. Am J Orthod Dentofacial Orthop.

[26] Kramer PF, Feldens CA, Ferreira SH, Spiguel MH, Feldens EG (2008). Dental anomalies and associated factors in 2- to 5-year old Brazilian children. Int J Paediatr Dent.

[27] Esenlik E, Sayın MO, Atilla AO, Ozen T, Altun C, Basak F (2009). Supernumerary teeth in a Turkish population. Am J Orthod Dentofacial Orthop.

[28] Kapdan A, Kustarci A, Buldur B, Arslan D, Kapdan A (2012). Dental anomalies in the primary dentition of Turkish children. Eur J Dent.

[29] Polder BJ, Van't Hof MA, Van der Linden FP, Kuijpers-Jagtman AM (2004). A meta-analysis of the prevalence of dental agenesis of permanent teeth. Community Dent Oral Epidemiol.

